# 超高效液相色谱-三重四极杆质谱法同时检测植物油中21种全氟及多氟烷基化合物

**DOI:** 10.3724/SP.J.1123.2024.01014

**Published:** 2024-08-08

**Authors:** Hao FENG, Wei ZHANG, Baoshan HE, Panpan LI, Shuqing GAO, Baoyuan GUO, Yongtan YANG

**Affiliations:** 1.河南工业大学粮油食品学院, 河南 郑州 450001; 1. College of Food Science and Technology, Henan University of Technology, Zhengzhou 450001, China; 2.国家粮食和物资储备局科学研究院, 北京 100037; 2. Academy of National Food and Strategic Reserves Administration, Beijing 100037, China

**Keywords:** 超高效液相色谱-三重四极杆质谱, 固相萃取, 全氟及多氟烷基化合物, 植物油, ultra-high performance liquid chromatography-triple quadrupole mass spectrometry (UHPLC-MS/MS), solid phase extraction (SPE), perfluorinated and polyfluoroalkyl substances (PFASs), plant oil

## Abstract

食用植物油是人们生活中重要的膳食组成,其质量安全关系到消费者健康。全氟及多氟烷基化合物(PFASs)可通过原料、加工过程、包装材料等多种途径污染植物油。因此建立植物油中PFASs的高灵敏、高准确分析方法对保障植物油质量安全至关重要。本研究建立了乙腈提取-固相萃取净化-超高效液相色谱-串联质谱法(UHPLC-MS/MS)同时检测植物油中全氟烷基羧酸、全氟烷基磺酸及多氟调聚磺酸三类21种PFASs的分析方法。实验对色谱分离条件、质谱检测参数进行优化,并考察了提取溶剂、固相萃取净化填料等样品前处理条件对样品回收率和净化效果的影响。确定植物油样品经乙腈直接提取,采用弱阴离子WAX SPE柱净化后,通过C18反相色谱柱进行分离;质谱检测器在电喷雾负离子模式下,采用多反应监测(MRM)模式进行检测,通过特征离子对及保留时间定性,采用内标法定量。结果表明,21种目标分析物在其相应的范围内线性关系良好,相关系数均≥0.995;方法的检出限和定量限分别为0.004~0.015 μg/kg和0.015~0.050 μg/kg,方法回收率为95.6%~115.8%,相对标准偏差为0.3%~10.9%(*n*=9)。本方法前处理操作简便,分析速度快,灵敏度高,抗干扰性强,稳定性高,适用于多种植物油中全氟烷基羧酸、全氟烷基磺酸及多氟调聚磺酸的快速检测分析。

## Abstract (en)

Edible plant oils are a key component of the daily human diet, and the quality and safety of plant oils are related to human health. Perfluorinated and polyfluoroalkyl substances (PFASs) are pollutants that can contaminate plant oil through the processing of raw materials or exposure to materials containing these substances. Thus, establishing a sensitive and accurate analytical method for the determination of PFASs is critical for ensuring the safety of plant oils. In this study, a method based on acetonitrile extraction and solid phase extraction purification combined with ultra-high performance liquid chromatography-triple quadrupole mass spectrometry (UHPLC-MS/MS) was developed for the simultaneous determination of 21 PFASs, including perfluorocarboxylic acids, perfluoroalkyl sulfonic acids, and fluorotelomer sulfonic acids, in edible plant oils. The chromatographic conditions and MS parameters were optimized, and the influences of the extraction solvents and purification method were systematically studied. Plant oil samples were directly extracted with acetonitrile and purified using a weak anion-exchange (WAX) column. The 21 target PFASs were separated on a reversed-phase C18 chromatographic column and detected using a triple quadrupole mass spectrometer with an electrospray ionization source. The mass spectrometer was operated in negative-ion mode. The target compounds were analyzed in multiple reaction monitoring (MRM) mode and quantified using an internal standard method. The results demonstrated that the severe interference observed during the detection of PFASs in the co-extracted substances was completely eliminated after the extraction mixture was purified using a WAX column. The 21 target PFASs showed good linearity in their corresponding ranges, with correlation coefficients greater than 0.995. The limits of detection (LODs) and limits of quantification (LOQs) of the method were in the range of 0.004-0.015 and 0.015-0.050 μg/kg, respectively. The recoveries ranged from 95.6% to 115.8%, with relative standard deviations (RSDs) in the range of 0.3%-10.9% (*n*=9). The established method is characterized by simple sample pretreatment, good sensitivity, high immunity to interferences, and good stability, rendering it suitable for the rapid analysis and accurate determination of typical PFASs in edible plant oils.

全氟及多氟烷基化合物(perfluorinated and polyfluoroalkyl substances, PFASs)是一类由人工合成的有机污染物^[1,2]^,其分子中的C-F键键能高,使其具有很高的化学稳定性和生物惰性^[3]^,被广泛用作涂料、农药、润滑剂等工业品及日常生活用品^[4,5]^。这些工业品和民用品在使用过程中和废弃后,其中的PFASs随之进入环境中^[2]^。目前,已在各种水源^[6,7]^、土壤^[8,9]^、沉积物^[10]^及空气^[11]^等环境介质中普遍检出各种PFASs。

越来越多的研究表明,PFASs可能会对生物体造成不良影响^[12]^,不仅会影响新陈代谢,而且具有潜在毒性,包括肝脏毒性^[13]^、心血管毒性^[14]^及生殖毒性^[15]^等。有研究表明,PFASs易在生物体中富集且可沿食物链放大^[16⇓-18]^。因此,PFASs被认为是持久性有机污染物^[19]^。其中,全氟辛酸(PFOA)、全氟辛基磺酸(PFOS)及其盐类和全氟己基磺酸(PFHxS)及其盐类已经被列入斯德哥尔摩公约^[20,21]^,世界各国也先后出台了各种措施限制PFASs类物质的生产和使用^[22]^。我国最新的“十四五”新污染物治理方案中,PFASs也是重点管控目标^[23]^。

PFASs可通过呼吸、接触和食物进入人体^[24]^。其中,食物是最主要的摄入途径^[16,25,26]^。食用植物油广泛用于日常食物烹调和食品加工^[27]^。但是,在食用油生产加工和储存过程中,可能会因接触包装产品、加工器具等而受到PFASs的污染。因此建立食用植物油中PFASs的检测方法十分必要。

食品中PFASs的含量极低^[28,29]^,一般采用高灵敏的气相色谱-串联质谱法^[30,31]^或液相色谱-串联质谱法^[32⇓⇓⇓-36]^检测。其中,气相色谱-串联质谱法主要用于检测挥发性PFASs^[30]^,当用于全氟烷基羧酸(PFCAs)和全氟烷基磺酸(PFSAs)等强极性化合物分析时,需要进行衍生化^[31]^,操作较复杂。液相色谱-串联质谱法的灵敏度和准确性高,尤其适用于痕量PFASs的直接分析^[32⇓⇓-35]^。但是,植物油中的主要成分甘油三酯和其他物质对质谱离子源的影响极大,严重制约了植物油中PFASs的检测分析。Ballesteros-Gómez等^[37]^和Noorlander等^[38]^均采用固相萃取柱净化的方法对食品及食用油中14种PFASs进行净化并使用液相色谱-串联质谱测定;徐睿等^[39]^采用减少样品用量,并使用有机溶剂进行稀释的方法结合液相色谱-质谱法测定食用油中7种PFASs;杨莉莉等^[40]^采用凝胶渗透色谱法(GPC)对食用油进行净化,之后采用液相色谱-串联质谱法测定18种PFASs。目前,文献中植物油中全氟酸类化合物检测方法主要针对PFCAs及部分PFSAs,而针对检测多氟调聚磺酸(FTSs)的方法鲜有报道。

本研究以植物油中11种全氟烷基羧酸、7种全氟烷基磺酸和3种多氟调聚磺酸为目标,采用乙腈提取-固相萃取净化的样品前处理方法,充分去除植物油共提取物的干扰,建立了同时测定21种PFASs的超高效液相色谱-三重四极杆质谱(UHPLC-MS/MS)分析方法,实现了植物油中多种PFASs的快速精准检测,可为保障植物油质量安全提供技术支撑。

## 1 实验部分

### 1.1 仪器、试剂与材料

6500Plus液相色谱-质谱仪(美国SCIEX公司); Fotector Plus全自动固相萃取仪、AutoEVA-60全自动平行浓缩仪(睿科集团(厦门)股份有限公司)。弱阴离子交换小柱(WAX, 200 mg/3 mL,上海安谱实验科技股份有限公司)。

色谱纯乙腈、甲醇、乙酸及乙酸铵、氨水(德国CNW公司);甲酸(美国赛默飞世尔科技有限公司);21种PFASs混合标准溶液:全氟丁酸(PFBA)、全氟戊酸(PFPeA)、全氟己酸(PFHxA)、全氟庚酸(PFHpA)、PFOA、全氟壬酸(PFNA)、全氟癸酸(PFDA)、全氟十一烷酸(PFUnA)、全氟十二烷酸(PFDoA)、全氟十三烷酸(PFTrTA)、全氟正十四烷酸(PFTeDA)、全氟-1-丁烷磺酸钾(PFBS)、全氟-1-戊烷磺酸钠(PFPeS)、PFHxS、全氟-1-庚烷磺酸钠(PFHpS)、PFOS、全氟-1-壬烷磺酸钠(PFNS)、全氟癸烷磺酸盐(PFDS)、1*H*,1*H*,2*H*,2*H*-全氟-1-己磺酸钠(4∶2 FTS)、1*H*,1*H*,2*H*,2*H*-全氟-1-辛烷磺酸钠(6∶2 FTS)、1*H*,1*H*,2*H*,2*H*全氟-1-癸烷磺酸钠(8∶2 FTS) (20 μg/mL,溶于甲醇-水(80∶20, v/v),美国Accustandard公司)。9种PFASs同位素内标混合标准溶液:^13^C_4_-PFBA、^13^C_2_ -PFHxA、^13^C_4_ -PFOA、^13^C_5_ -PFNA、^13^C_2_ -PFDA、^13^C_2_ -PFUnA、^13^C_2_ -PFDoA、^18^O_2_ -PFHxS、^13^C_4_ -PFOS (2 μg/mL,溶于甲醇,加拿大Wellington公司)。

植物油样品(大豆油、花生油、橄榄油、菜籽油、玉米油、芝麻油、油茶籽油、亚麻籽油)均为市售产品。

### 1.2 标准溶液配制

精确移取21种PFASs混合标准溶液,用甲醇稀释,配制质量浓度为25 ng/mL的混合标准工作液;内标混合标准液以甲醇进行稀释,配制质量浓度为30 ng/mL的内标混合标准工作液。所有工作液在-18 ℃下避光保存。

将21种PFASs混合标准工作液用甲醇稀释,并加入定量内标混合标准工作液,配制成质量浓度分别为0.1、0.2、0.5、1.0、2.0、5.0、10.0、20.0 ng/mL的系列标准溶液,内标的质量浓度均为3 ng/mL。

### 1.3 样品前处理

准确称取5.00 g (精确至0.01 g)植物油样品于50 mL具塞聚丙烯离心管中,加入50 μL混合内标标准工作液(30 ng/mL),随后加入10 mL乙腈,充分振荡3 min,静置1 min,于7000 r/min下离心3 min,取上清层8 mL转移至15 mL玻璃试管中;于室温氮气流下浓缩至近干,使用1 mL甲醇复溶,采用WAX小柱净化。小柱采用3 mL甲醇和5 mL 2%甲酸水溶液依次活化,将待净化样品转移上样后,依次采用2 mL 2%甲酸水溶液、2 mL纯水、5 mL甲醇进行淋洗,最后采用10 mL 0.25%氨水甲醇溶液洗脱。收集洗脱液至15 mL试管中,在氮气流下浓缩至干后,用0.5 mL甲醇复溶,采用UHPLC-MS/MS测定。

### 1.4 UHPLC-MS/MS条件

#### 1.4.1 色谱条件

色谱柱:ZORBAX Eclipse Plus C18 (50 mm×2.1 mm, 1.8 μm);柱温:35 ℃;进样量:2 μL;流动相A: 0.1%乙酸水溶液;流动相B: 0.1%乙酸甲醇溶液;流速:0.3 mL/min。梯度洗脱程序:0~1 min, 10%B; 1~11 min, 10%B~95%B; 11~14 min, 95%B; 14~14.5 min, 95%B~10%B; 14.5~17 min, 10%B。

#### 1.4.2 质谱条件

采用电喷雾离子源(ESI),负离子模式检测。喷雾电压:-4500 V;气帘气(CUR)压力: 241 kPa;雾化气(GS 1)压力:310 kPa;加热辅助气(GS 2)压力:207 kPa;离子源温度(TEM): 300 ℃;去簇电压(DP): -20 V。采用多反应监测(MRM)模式进行检测。21种目标化合物和9种内标物的监测离子对及碰撞能量(CE)如表1所示。

**表1:** 21种PFASs和9种内标物的保留时间和质谱参数

Compound	Type	Retention time/min	Parent ion (m/z)	Daughter ions (m/z)	CEs/eV	IS
Perfluoro-n-butanoic acid (PFBA)	PFCAs	4.05	213.0	168.9^*^	-11	^13^C_4_-PFBA
Perfluoro-n-pentanoic acid (PFPeA)	PFCAs	6.74	263.0	218.9^*^	-12	^13^C_2_ -PFHxA
Perfluoro-n-hexanoic acid (PFHxA)	PFCAs	8.10	313.0	269.0^*^/119.0	-12/-26	^13^C_2_ -PFHxA
Perfluoro-n-heptanoic acid (PFHpA)	PFCAs	9.01	363.0	319.0^*^/169.0	-13/-22	^13^C_4_ -PFOA
Perfluoro-n-octanoic acid (PFOA)	PFCAs	9.70	413.0	369.0^*^/169.0	-16/-25	^13^C_4_ -PFOA
Perfluoro-n-nonanoic acid (PFNA)	PFCAs	10.33	463.0	419.0^*^/219.0	-15/-25	^13^C_5_ -PFNA
Perfluoro-n-decanoic acid (PFDA)	PFCAs	10.87	513.0	469.0^*^/219.0	-15/-25	^13^C_2_ -PFDA
Perfluoro-n-undecanoic acid (PFUnA)	PFCAs	11.36	563.0	519.0^*^/269.0	-16/-27	^13^C_2_ -PFUnA
Perfluoro-n-dodecanoic acid (PFDoA)	PFCAs	11.84	613.0	569.0^*^/169.0	-17/-37	^13^C_2_ -PFDoA
Perfluoro-n-tridecanoic acid (PFTrTA)	PFCAs	12.31	663.0	619.0^*^/169.0	-17/-36	^13^C_2_ -PFDoA
Perfluoro-n-tetradecanoic acid (PFTeDA)	PFCAs	12.79	713.0	669.0^*^/169.0	-21/-36	^13^C_2_ -PFDoA
Potassium perfluoro-1-butanesulfonate (PFBS)	PFSAs	7.23	299.0	99.0/80.0^*^	-65/-70	^18^O_2_ -PFHxS
Sodium perfluoro-1-pentanesulfonate (PFPeS)	PFSAs	8.30	349.0	99.0/80.0^*^	-72/-85	^18^O_2_ -PFHxS
Potassium perfluoro-1-hexanesulfonate (PFHxS)	PFSAs	9.08	399.0	99.0/80.0^*^	-80/-99	^18^O_2_ -PFHxS
Sodium perfluoro-1-heptanesulfonate (PFHpS)	PFSAs	9.75	449.0	99.0/80.0^*^	-102/-116	^13^C_4_ -PFOA
Potassium perfluoro-1-octanesulfonate (PFOS)	PFSAs	10.32	499.0	99.0/80.0^*^	-103/-103	^13^C_4_ -PFOS
Sodium perfluoro-1-nonanesulfonate (PFNS)	PFSAs	10.87	549.0	99.0/80.0^*^	-129/-129	^13^C_2_ -PFDA
Sodium perfluoro-1-decanesulfonate (PFDS)	PFSAs	11.35	599.0	99.0/80.0^*^	-120/-130	^13^C_2_ -PFUnA
Sodium 1H,1H,2H,2H-perfluoro-1-hexanesulfonate (4∶2 FTS)	FTSs	7.99	327.0	307.0^*^/81.0	-27/-66	^13^C_2_ -PFHxA
Sodium 1H,1H,2H,2H-perfluoro-1-octanesulfonate (6∶2 FTS)	FTSs	9.67	427.0	407.0^*^/81.0	-36/-81	^18^O_2_ -PFHxS
Sodium 1H,1H,2H,2H-perfluoro1-decanesulfonate (8∶2 FTS)	FTSs	10.90	527.0	507.0^*^/81.0	-39/-102	^13^C_2_ -PFDA
^13^C_4_-PFBA	PFCAs	4.05	216.0	171.0^*^	-11	-
^13^C_2_ -PFHxA	PFCAs	8.10	314.0	269.0^*^	-12	-
^13^C_4_ -PFOA	PFCAs	9.70	417.0	372.0^*^	-16	-
^13^C_5_ -PFNA	PFCAs	10.33	468.0	423.0^*^	-15	-
^13^C_2_ -PFDA	PFCAs	10.87	515.0	470.0^*^	-15	-
^13^C_2_ -PFUnA	PFCAs	11.36	565.0	520.0^*^	-16	-
^13^C_2_ -PFDoA	PFCAs	11.84	615.0	570.0^*^	-17	-
^18^O_2_ -PFHxS	PFSAs	9.08	403.0	84.0^*^	-99	-
^13^C_4_ -PFOS	PFSAs	10.32	503.0	80.0^*^	-103	-

CE: collision energy; * quantitative ion; PFCAs: perfluorocarboxylic acids; PFSAs: perfluoroalkyl sulfonic acids; FTSs: fluorotelomersulfonic acid.

## 2 结果与讨论

### 2.1 质谱条件优化

采用三重四极杆质谱法进行分析时,每个化合物需要确定两对特征离子对,以提高定性和定量的准确性。PFCAs、PFSAs、FTSs均为强极性酸性化合物,在质谱中采用负离子模式检测,采用[M-H]^-^作为检测离子对的母离子。之后,采用子离子扫描方式得到母离子的特征产物离子,并且对特征碰撞能量进行优化以获得响应最高的子离子。根据质谱信息,PFCAs(C*_n_*HF_2_*_n_*_-1_O_2_)的碎片离子为[C*_n-_*_1_F_2_*_n_*_-1_]^-^,是准分子离子[M-H]^-^进一步失去CO_2_的产物[M-COOH]^-^,以及后续断裂多个二氟代亚甲基(*n*CF_2_)所形成的负离子[M-COOH-*n*CF_2_]^-^。对于PFSAs(C*_n_*HF_2_*_n_*_+1_O_3_S),其主要碎片离子为三氧化硫负离子[SO_3_]^-^及氟代磺酸根离子[FSO_3_]^-^。对于*n*∶2 FTSs (C*_n_*_+2_H_5_F_2_*_n_*_+1_SO_3_),其主要碎片离子为丢失2个H和1个F的负离子[C*_n_*_+2_H_3_F_2_*_n_*SO_3_]^-^及磺酸根负离子[HSO_3_]^-^。对于每个目标分析物,选择响应最好的两对离子对作为定量离子对和定性离子对。其中,响应最好的离子对用于定量,响应稍差的离子对用于辅助定性。但是,对于PFBA和PFPeA,由于碳链短,均只有一个具有较高响应的碎片离子,分别为[C_3_F_7_]^-^和[C_4_F_9_]^-^,因此对这两个化合物,仅可以监测到一对离子对。每个目标分析物在多反应监测模式下的离子对及最优碰撞能量见表1。

### 2.2 色谱条件优化

PFASs分子中具有亲脂性烷基碳链与亲水性酸性官能团,因酸性官能团种类及烷基碳链长度的差异,其在色谱分离上的表现不同。反相C18色谱柱常用于PFASs的分离分析。本研究比较了ZORBAX Eclipse Plus C18(50 mm×2.1 mm, 1.8 μm)、Accucore C18(100 mm×3.0 mm, 2.6 μm)、SUPELCOSIL C18(150 mm×4.6 mm, 3 μm) 3种不同规格的C18色谱柱。结果表明,21种目标分析物在3种色谱柱上均可较好分离,其中使用较短的ZORBAX Eclipse Plus C18柱,所有目标化合物都具有较高的响应值,因此选用该色谱柱。

使用水-甲醇为流动相时,目标分析物的色谱峰发生严重偏倚(图1a)。在水中和甲醇中均加入0.1%乙酸后,峰形得到很好的改善(图1b);而使用乙酸铵(2 mmol/L)为添加剂时,所有目标物的响应均下降,其中3种多氟调聚磺酸(4∶2 FTS、6∶2 FTS、8∶2 FTS)响应下降较多(56.7%~62.2%)(图1c),这与文献[41]报道的乙酸铵对质谱检测具有抑制作用的结论一致。在水相中同时加入乙酸铵和乙酸后,目标物响应并未改善(图1d)。此外,随着流动相中乙酸添加量(0.1%、0.2%、0.5%)的增加,所有目标物的保留时间缩短,但响应没有明显变化。而使用含有0.1%乙酸的乙腈为有机相时,长链全氟羧酸(PFUnA、PFTrTA、PFTeDA)的色谱峰峰形极差(图1e),不适于分析。因此,本研究选择0.1%乙酸水溶液和0.1%乙酸甲醇溶液作为流动相体系。

**图1:**
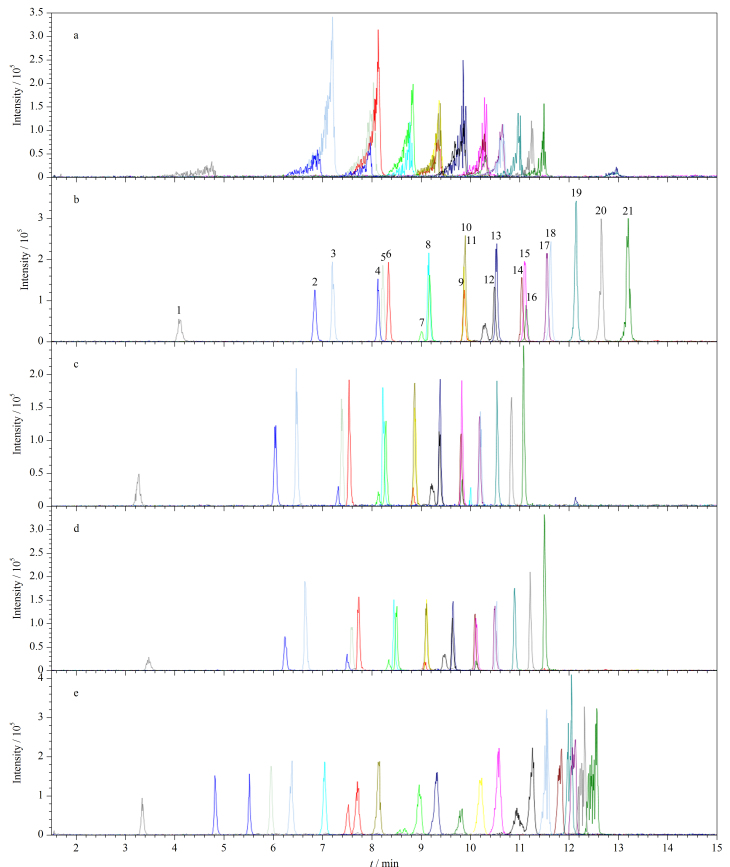
采用不同流动相体系时21种PFASs的提取离子流色谱图

### 2.3 样品前处理条件优化

#### 2.3.1 提取溶剂的优化

不同基质中PFASs的含量往往很低,需要使用提取溶剂进行提取并浓缩。PFASs均为酸性化合物,通常使用甲醇或乙腈等极性有机溶剂进行提取。本研究选取甲醇、乙腈、1%甲酸乙腈、1%乙酸乙腈、0.25%氨水乙腈作为提取溶剂对食用植物油中的PFASs进行提取(见图2)。结果表明:使用甲醇和乙腈均可较好地提取植物油中的PFCAs、PFSAs、FTSs,回收率为79.7%~118.3%。其中,乙腈提取的效果较甲醇提取的效果更好,因此选用乙腈作为提取溶剂。随后对不同改性剂进行比较,结果发现,在乙腈中添加甲酸和乙酸对21种目标分析物的提取回收率没有明显影响,但使用氨水碱化的乙腈时,21种目标分析物的回收率严重降低。其原因可能是因为氨水与全氟或多氟酸类化合物形成离子对,从而降低其在乙腈中的溶解度。因此,本研究采用乙腈作为提取溶剂对植物油中PFASs进行提取。

**图2:**
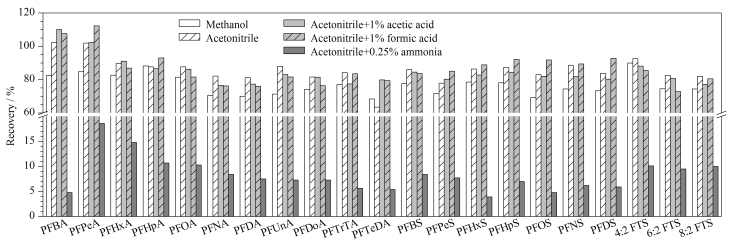
采用不同提取溶剂时21种PFASs的回收率

#### 2.3.2 净化条件的优化

植物油样品净化一般采用凝胶渗透色谱法、固相萃取法和分散固相萃取法。其中,GPC需要使用昂贵的仪器,费时费力,并且需要大量有机溶剂;而分散固相萃取法的净化效果有限。本研究采用固相萃取法进行净化,以花生油为代表样品,比较了采用不同填料(弱阴离子交换、强阴离子交换(SAX)、*N*-丙基乙二胺(PSA)、亲水亲脂平衡(HLB)、C18和活性炭(Envi-Carb))固相萃取柱(200 mg/3 mL)的净化效果(见图3)。结果显示:使用HLB、C18和Envi-Carb填料,所有PFASs的回收率为0.3%~10.9%;使用SAX填料净化后,PFCAs和FTSs的回收率为67.1%~108.4%,但PFSAs的回收率较低,为0~4.8%。虽然WAX填料和PSA填料均可以有效净化植物油样品中的共提取物,但WAX填料的净化效果更好,因此选用弱阴离子交换固相萃取柱。

**图3:**
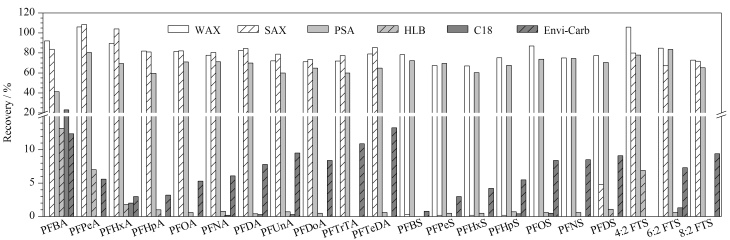
采用不同净化填料时21种PFASs的回收率

### 2.4 方法学评价

#### 2.4.1 基质效应

植物油样品中的脂类物质会对质谱分析造成严重干扰,容易产生基质效应。本研究采用花生油为植物油代表样品,配制空白基质标准溶液。采用公式(1)评价基质效应:


(1) ME=*A/B*×100%

其中,*A*为基质匹配标准曲线的斜率,B为溶剂标准曲线的斜率。当ME大于120%时,认为存在较强的基质增强效应,ME小于80%时,认为存在较强的基质抑制效应,ME为80%~120%时,则认为基质效应可忽略。结果如表2所示,21种PFASs的ME为93.9%~111.3%,表明经净化后,基本不存在基质效应。因此,本研究通过溶剂标准曲线定量。

**表2:** 21种PFASs的回归方程、线性范围、相关系数、检出限、定量限和基质效应

Compound	Regression equation	Linear range/(μg/L)	R^2^	LOD/(μg/kg)	LOQ/(μg/kg)	ME/%
PFBA	y=0.96544x+0.07542	0.5-20	0.996	0.015	0.050	102.1
PFPeA	y=0.63301x+0.01894	0.5-20	0.998	0.014	0.050	111.3
PFHxA	y=1.31366x+0.01116	0.5-20	0.996	0.013	0.043	104.8
PFHpA	y=0.76716x+0.00292	0.3-20	0.998	0.009	0.030	109.0
PFOA	y=0.92176x+0.02257	0.4-20	0.997	0.012	0.039	103.2
PFNA	y=1.10739x+0.02129	0.3-20	0.996	0.009	0.030	97.4
PFDA	y=0.90682x+0.01359	0.2-20	0.997	0.006	0.020	94.6
PFUnA	y=0.85528x+0.01771	0.2-20	0.996	0.005	0.018	107.4
PFDoA	y=0.89598x+0.02313	0.2-20	0.997	0.004	0.015	100.4
PFTrTA	y=0.69193x+0.00830	0.2-20	0.997	0.005	0.018	96.5
PFTeDA	y=0.69582x+0.00807	0.2-20	0.997	0.006	0.020	93.9
PFBS	y=1.19856x+0.04642	0.4-20	0.996	0.011	0.036	99.4
PFPeS	y=1.13025x+0.01930	0.4-20	0.998	0.013	0.039	108.9
PFHxS	y=1.06398x-0.00567	0.4-20	0.999	0.012	0.036	100.3
PFHpS	y=0.70470x+0.01110	0.3-20	0.995	0.007	0.024	101.3
PFOS	y=0.60818x+0.00349	0.5-20	0.996	0.015	0.050	97.4
PFNS	y=0.43723x+0.00929	0.4-20	0.998	0.009	0.033	100.7
PFDS	y=0.45889x+0.00763	0.2-20	0.995	0.006	0.020	101.7
4∶2 FTS	y=1.07666x+0.00570	0.3-20	0.997	0.009	0.030	101.0
6∶2 FTS	y=0.76250x+0.00543	0.3-20	0.997	0.010	0.030	95.2
8∶2 FTS	y=0.47753x+0.00637	0.3-20	0.999	0.011	0.030	104.6

*y*: peak area ratio of the target to internal standard; *x*: mass concentration ratio of the target to internal standard.

#### 2.4.2 线性关系、检出限与定量限

配制质量浓度分别为0.1、0.2、0.5、1、2、5、10、20 μg/L的系列标准混合溶液,并在优化的实验条件下进行分析,以内标法进行定量。以待测物和内标物的质量浓度比为横坐标、色谱峰面积比为纵坐标进行线性回归分析,绘制标准曲线。结果显示,21种PFASs在各自范围内线性关系良好,所有化合物在线性范围内的线性相关系数(*R*^2^)均≥0.995。

向花生油空白样品中添加低浓度混合标准溶液后进行分析。分别以信噪比为3(*S/N*=3)和*S/N*=10确定各目标分析物的检出限(LOD)和定量限(LOQ)。结果如表2所示,21种PFASs的检出限和定量限分别为0.004~0.015 μg/kg和0.015~0.050 μg/kg。

#### 2.4.3 回收率和精密度

取5.00 g花生油空白样品3份,分别向其中添加不同浓度的混合标准溶液,配制成0.05、0.2和1.0 μg/kg 3个水平的空白添加样品,对其进行样品前处理和分析,采用内标法定量,每个水平测定9次,计算方法回收率和相对标准偏差,以评价方法的准确性和精密度。结果如表3所示,21种目标分析物的平均回收率为95.6%~115.8%, RSD为0.3%~10.9%。结果表明分析方法具有较好的准确度和精密度。

**表3:** 21种PFASs在植物油样品中的加标回收率和相对标准偏差(*n*=9)

Compound	Spiked recoveries (RSDs)/%		
	0.05 μg/kg	0.2 μg/kg	1.0 μg/kg
PFBA	108.7 (3.6)	101.2 (1.8)	99.7 (2.1)
PFPeA	107.4 (5.7)	106.9 (4.6)	99.5 (3.4)
PFHxA	96.9 (9.2)	99.7 (4.8)	101.3 (2.9)
PFHpA	106.9 (6.9)	105.0 (5.3)	106.7 (2.9)
PFOA	107.2 (10.9)	112.0 (8.3)	104.4 (4.5)
PFNA	101.6 (5.7)	109.3 (2.2)	101.5 (5.3)
PFDA	109.4 (7.3)	112.8 (4.6)	102.2 (4.0)
PFUnA	113.2 (5.7)	108.8 (3.7)	105.7 (4.1)
PFDoA	98.2 (4.2)	106.4 (6.9)	109.0 (2.4)
PFTrTA	114.2 (8.4)	111.8 (5.4)	102.0 (5.7)
PFTeDA	101.4 (4.2)	100.2 (6.0)	97.4 (9.3)
PFBS	98.5 (9.3)	107.3 (4.3)	103.9 (3.3)
PFPeS	95.6 (2.9)	112.7 (6.8)	97.5 (4.3)
PFHxS	112.3 (2.9)	101.1 (6.3)	98.9 (0.5)
PFHpS	97.3 (3.9)	97.4 (3.8)	101.1 (2.4)
PFOS	109.7 (0.3)	105.7 (2.9)	100.7 (3.1)
PFNS	105.5 (8.5)	103.2 (4.5)	102.8 (2.3)
PFDS	99.9 (8.3)	104.5 (8.0)	107.5 (3.4)
4∶2 FTS	97.6 (10.0)	101.1 (6.3)	96.8 (4.9)
6∶2 FTS	110.7 (1.9)	99.4 (4.6)	96.5 (1.3)
8∶2 FTS	115.8 (8.8)	114.4 (2.4)	104.2 (1.4)

### 2.5 实际样品检测

采用建立的分析方法,对市场上43种植物油样品进行测定。植物油样品包括2种大豆油、9种花生油、21种菜籽油、1种玉米油、3种芝麻油、4种油茶籽油、2种亚麻籽油和1种橄榄油。结果显示,在多数植物油中未检出PFASs,仅在少量样品中检出了6∶2 FTS、PFHpS和PFOS(见表4)。其中,在3个菜籽油中检出PFHpS,含量分别为0.3053 ng/g、0.0720 ng/g和<LOQ。在2个亚麻籽油中检出6∶2 FTS,但含量<LOQ。在2个菜籽油和1个芝麻油中检出PFOS,其中芝麻油中PFOS的含量为0.1396 ng/g,而菜籽油中PFOS的含量仅高于检出限。此外,在2个菜籽油中筛查出PFPeA,由于该化合物仅有一对监测离子对,因此需要进一步进行确证。

**表4:** 植物油样品中PFASs的检测结果

Sample	6∶2 FTS	PFHpS	PFOS	PFPeA
Linseed oil-1	<LOQ	ND	ND	ND
Linseed oil-2	<LOQ	ND	ND	ND
Sesame oil-1	ND	ND	<LOQ	ND
Sesame oil-2	ND	ND	0.1396	ND
Rapeseed oil-1	ND	ND	ND	<LOQ
Rapeseed oil-2	ND	ND	ND	<LOQ
Rapeseed oil-3	ND	0.3053	<LOQ	ND
Rapeseed oil-4	ND	0.0720	ND	ND
Rapeseed oil-5	ND	<LOQ	ND	ND

## 3 结论

本研究建立了植物油中21种PFCAs、PFSAs和FTSs同时分析的测定方法。方法前处理简单,提取效率高,通过WAX固相萃取柱净化,可以有效去除基质对分析的干扰。方法具有较高的灵敏度、准确度和精密度,抗干扰能力强,分析速度快,适用于植物油中痕量PFASs的分析。采用所建立的分析方法对我国市场上主要的植物油样品进行分析,发现仅在少量样品中检出痕量PFASs,表明我国植物油尚未受到PFASs的污染。尽管如此,仍需要对植物油进行持续监测,以保障其质量安全。
